# Influence of Anion and Cation Structure of Ionic Liquids on Carboxylic Acids Extraction

**DOI:** 10.3389/fchem.2019.00117

**Published:** 2019-03-14

**Authors:** Ján Marták, Štefan Schlosser

**Affiliations:** Institute of Chemical and Environmental Engineering, Faculty of Chemical and Food Technology, Slovak University of Technology, Bratislava, Slovakia

**Keywords:** extraction, carboxylic acids, ionic liquids, influence of structure, model, L/L equilibrium

## Abstract

A recently proposed new mechanism and a model of reactive extraction of carboxylic acids by hydrophobic ionic liquids (ILs) was tested on five systems from published as well as from new equilibrium data on liquid-liquid extraction of butyric and lactic acids (BA and LA) from aqueous solutions. Two phosphonium and one ammonium ILs were used. The model describes experimental data for all systems with a good fit. The mechanism of acid extraction by ILs is very similar for all tested systems. This indicates a more general validity of the developed model. The model allows deeper understanding of regularities in carboxylic acid extraction by hydrophobic ILs. Stability constants of the first acid-IL bonds are by one to three orders of magnitude higher compared to that of acid-acid bonds. Values of stability constants related to two acid-IL bonds are sensitive to a cation and anion structure while stability constants for acid-acid bonds more distant from polar head of IL are not sensitive to IL structure. The stability constants of acid-IL bonds for LA and phosphonium ILs are by more than one order of magnitude lower compared to those for BA and are not influenced with an anion structure. The value of stability constant for the first BA-IL bond is for phosphonium IL with a decanoate anion only one third of those for IL with a phosphinate anion. Differences in the stability of acid-IL bonds for BA and LA can be attributed to hydrophobic interactions which almost do not occur in LA extraction. Ammonium IL also forms a less stable BA-IL bond than the phosphonium IL with the same phosphinate anion. A less stable BA-IL bond can favor the higher recovery of volatile acid from the solvent by vacuum evaporation where free acid is separated instead of acid salts as in classical processes what is a great advantage.

## Introduction

Phosphonium and ammonium ionic liquids (ILs) are effective solvents for extraction of carboxylic acids (Schlosser et al., 2018) which could be of interest as platform chemicals produced from renewable resources (Bozell and Petersen, 2010; Schlosser and Blahušiak, 2011). The mechanism of carboxylic acids extraction by hydrophobic ILs is rather complex (Sprakel and Schuur, 2019). Several phenomena participate in this process, e.g., competitive extraction of acid and water, coextraction of acid and water, aggregation and segregation in ILs, formation of reverse micelles in the organic phase, synergistic effect between the IL cation and anion, etc. (Schlosser et al., 2018). The structure of an anion and cation strongly influences the extraction performance (Schlosser et al., 2018). A new mechanism and model of carboxylic acids extraction by hydrophobic ILs was proposed in paper (Marták and Schlosser, 2016) and tested on butyric acid (BA) extraction with a good fit.

The aim of this work was to test a new model of carboxylic acids extraction by hydrophobic ILs (Marták and Schlosser, 2016) on data for five systems: earlier published (Marták and Schlosser, 2007; Blahušiak et al., 2013) and new equilibrium data on liquid-liquid extraction of butyric and lactic acids (BA and LA) by two phosphonium and one ammonium ILs from aqueous solutions.

## Theory

The most important characteristics of the new extraction mechanism and model of liquid-liquid equilibrium developed in paper (Marták and Schlosser, 2016) are presented in this chapter with some actualizations enabling its more general applicability. A more detailed description of the model is in Marták and Schlosser (2016).

Reactive extraction of monocarboxylic acid, AH, by IL takes place by the formation of (*p*, 1) complexes containing *p* molecules of acid and one ion pair of IL. In water saturated ILs with phosphinate and carboxylate anions water is associated around two H-bonding sites located on the carboxylate or phosphinate oxygens of IL anions. The H-bonds as an important and very general phenomenon in ILs was discussed in Hunt (2017). It is assumed that after the addition of AH, an acid-IL complex (*p*, 1) is formed by the **competitive mechanism** resulting in the replacement of water surrounding these H-bonding sites with AH according to the following equation:

(1) $$\begin{array}{l} \begin{array}{ccc} \text{AH} & + & \bar{{\left(\text{AH}\right)}_{p-1}{\left({\text{H}}_{2}\text{O}\right)}_{k_{p-1}}\text{IL}}⇄\bar{{\left(\text{AH}\right)}_{p}{\left({\text{H}}_{2}\text{O}\right)}_{k_{p}}\text{IL}} \end{array}\\ \;\begin{array}{cc} + & (k_{p-1}-k_{p}){\text{H}}_{2}\text{O }\;\;K_{p}=\frac{c_{p}}{c_{\text{F}}c_{p-1}} \end{array} \end{array}$$

For *p* = 1 in Equation (1), *c*_0_ is the concentration of water saturated acid-free IL in the organic phase. In the case of LA extraction by all tested ILs and BA extraction by ILs with phosphinate anions *k*_*p*_ for *p* ≥ 2 is equal to zero so that in the complexes (*p*, 1) with these *p* there is no water directly associated with IL. The same follows also for BA extraction by phosphonium decanoate with the exception that *p* ≥ 3.

Once the constant *k*_*p*_ is equal to zero, binding of the next acid does not compete with water, but it forms H-bond with the acid already contained in complex. Therefore, the complexes with higher *p* are formed by a **non-competitive mechanism**. In the studied systems, this follows typically for complexes with *p* > 2 except for the system with BA and IL with decanoate anion where it is for *p* > 3. For the non-competitive mechanism, the following equation can be written

(2) $$\begin{array}{c} \text{AH}+\bar{{\text{(AH)}}_{p-1}\text{IL}}⇄\bar{{\text{(AH)}}_{p}\text{IL}}\;\;K_{p}=\frac{c_{p}}{c_{\text{F}}c_{p-1}} \end{array}$$

**Figure 1:**
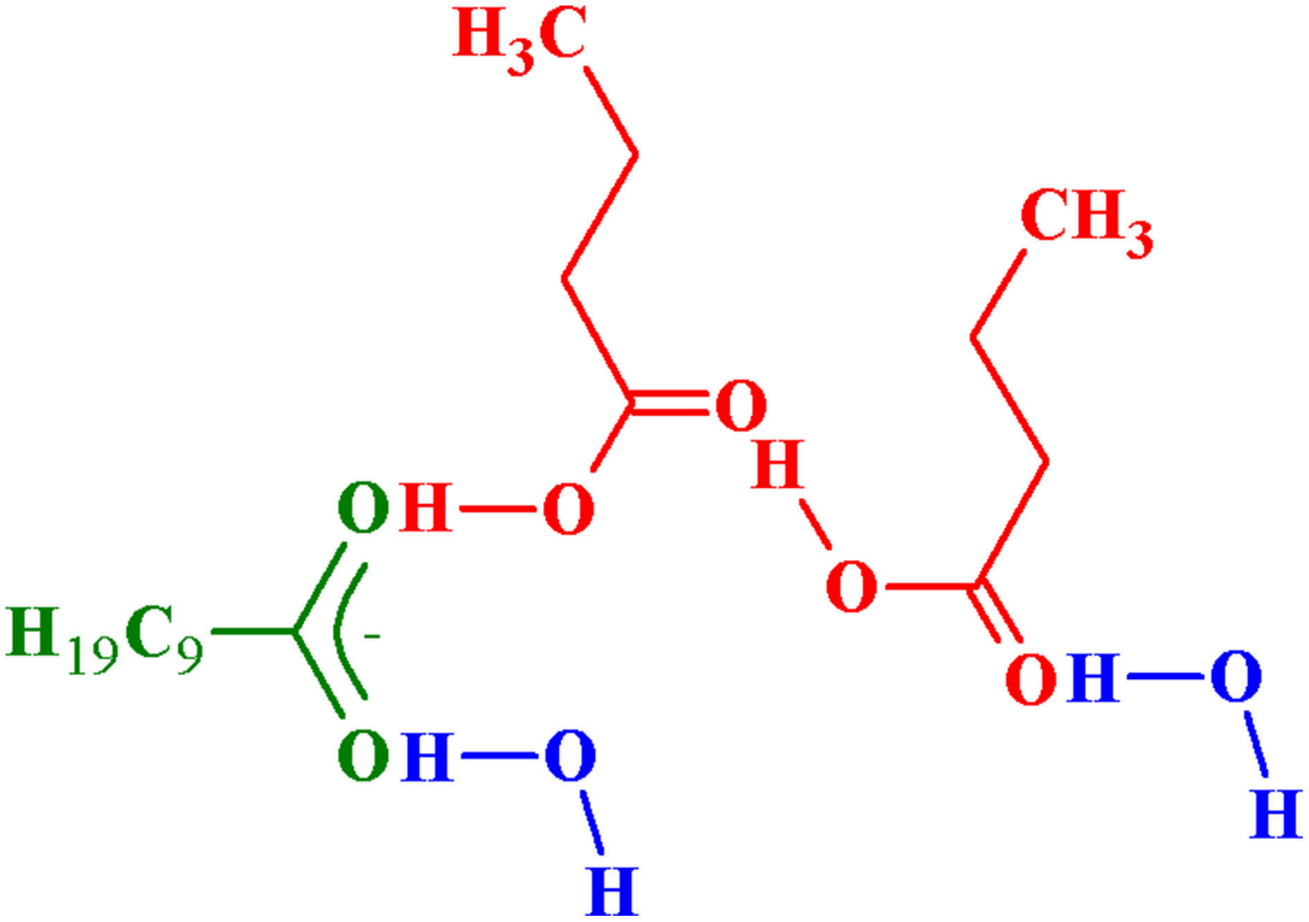
Proposed structural formula of BA-[C_14_C_6_C_6_C_6_P^+^][C_9_COO^−^] complex (2, 1).

*K*_*p*_ in Equations (1, 2) are the stability constants (equilibrium constant) characterizing the stability of the bond between acid and IL in complexes (1, 1) and (2, 1), and between two acids in complexes with *p* > 2. They are defined in terms of molar concentrations. For system with BA and decanoate IL one acid-acid bond is proposed also in complex (2, 1) as shown in Figure 1. The proposed structural formulas of (*p*, 1) complexes were published in our previous works (Marták and Schlosser, 2016, 2017). For more hydrophobic acids, e. g. butyric acid (BA), the acid-IL and acid-acid hydrophobic interactions between hydrocarbon chains occur. In such case *K*_*p*_ is lumped constant including also van der Waals interactions.

Using Equations (1, 2), molar ratio of the (*p*, 1) complex to IL is:

(3) $$\begin{array}{c} u_{p}=\frac{n_{p}}{n_{\text{I}}}=\frac{\left(\prod_{i=1}^{p}K_{i}\right)c_{\text{F}}^{p}}{1+\sum_{p=1}^{n}\left(\prod_{i=1}^{p}K_{i}\right)\;c_{\text{F}}^{p}} \end{array}$$

Loading of IL by AH is defined as the molar ratio of reactively extracted acid and IL:

(4) $$\begin{array}{c} z_{\text{AH}}=\frac{n_{\text{S}}}{n_{\text{I}}}=\sum_{p=1}^{n}pu_{p} \end{array}$$

From Equations (3, 4) follows that the loading is independent of the IL concentration. The same applies to all monobasic acids. The values of *K*_*p*_ systematically decrease with the increasing *p*. For the mathematical expression of this dependence, an exponential decay according to the following empirical formula has been suggested:

(5) $$\begin{array}{c} \ln\;K_{p}=A+Be^{Cp} \end{array}$$

where *A, B*, and *C* are empirical parameters. The selection of this empirical dependence is elucidated in the previous work (Marták and Schlosser, 2016).

Fitting of equilibrium data from the AH extraction was done using *z*_AH_ as a function of *c*_F_, which is the concentration of undissociated AH in the aqueous phase. The relation between the analytical AH concentrations in the aqueous phase *c*_aF_ and *c*_F_ can be derived using p*K*_*a*_ of AH and equilibrium pH as follows:

(6) $$c_{\text{F}}=\frac{c_{\text{aF}}}{10^{{\text{(pH}}_{\text{F}}-\text{p}K_{a})}+1}$$

p*K*_*a*_ = 4.821 at the temperature of 298 K was used for butyric acid (Partanen, 2004) and p*K*_*a*_ = 3.86 for lactic acid (Dawson et al., 1986).

Apart from water directly associated with IL which competes with acid according to Equation (1) another type of water is extracted to the organic phase together with acid by **coextraction mechanism**. In other words, the extracted acid is hydrated. Therefore, the total equilibrium loading of IL by water in the organic phase was defined as a sum of loadings by water directly associated with the IL which competes with AH, and water co-extracted (associated) with the acid

(7) $$\begin{array}{c} z_{\text{W}}=z_{\text{W},\text{ comp}}+z_{\text{W},\text{ coext}} \end{array}$$

where

(8) $$\begin{array}{c} z_{\text{W},\text{ comp}}=k_{0}u_{\text{I},\text{ AHfree}}+k_{1}u_{1}+k_{2}u_{2} \end{array}$$

(9) $$\begin{array}{c} z_{\text{W},\text{ coext}}=K_{\text{W},\text{ coext}}z_{\text{AH}} \end{array}$$

and

(10) $$\begin{array}{c} u_{\text{I},\;\text{AHfree}}=\frac{n_{\text{I},\;\text{AHfree}}}{n_{\text{I}}}=\frac{1}{1+\sum_{p=1}^{n}\left(\prod_{i=1}^{p}K_{i}\right)c_{F}^{p}} \end{array}$$

is the molar ratio of IL not associated with AH and the total IL. Experimental results indicated that the amount of coextracted water is linearly dependent on the amount of extracted acid. This is expressed by Equation (9) where *K*_W,coext_ is the water coextraction constant. Fitting of equilibrium data on water extraction was done using *z*_W_ as a function of *c*_F_ by combining (Equations 7–10, 3).

Coefficients *k*_*p*_ represent only water extracted by the competitive mechanism (see Equation 1). All water associated with IL and AH in complexes (1, 1) and (1, 2) (Marták and Schlosser, unpublished manuscript) can be calculated as follows

(11) $$\begin{array}{c} k_{1,\text{ Wtot}}=k_{1}+K_{\text{W},\text{ coext}} \end{array}$$

(12) $$\begin{array}{c} k_{2,\text{ Wtot}}=k_{2}+2K_{\text{W},\text{ coext}} \end{array}$$

Thus, the overall mechanism of simultaneous extraction of AH and water by IL can be divided into three sub-mechanisms:

- Competitive extraction of acid and water
- Non-competitive mechanism of AH extraction
- Co-extraction of water with AH

## Materials and Methods

**Figure 2:**
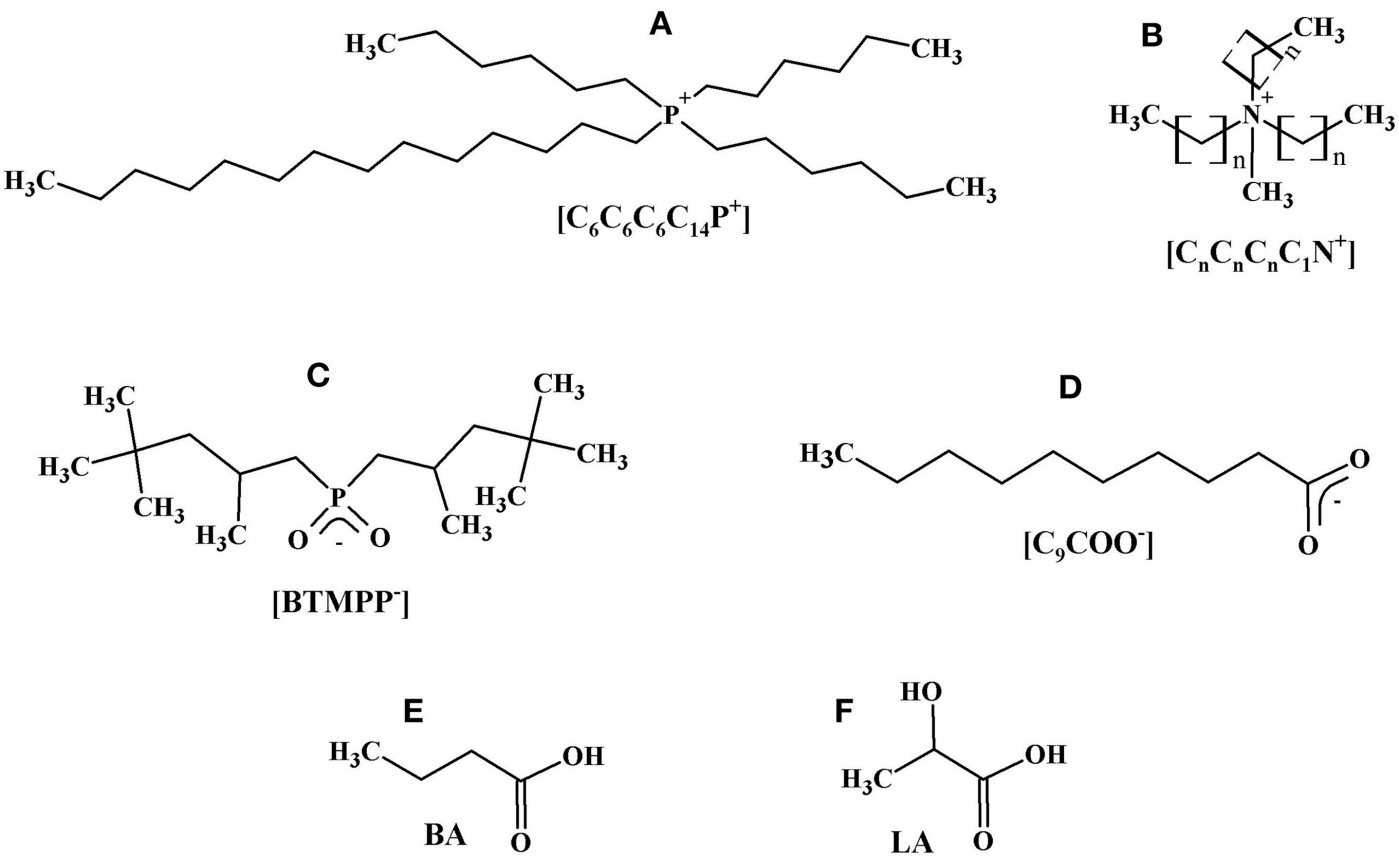
Structural formulas of tetradecyltrihexylphosphonium **(A)** trialkylmethylammonium **(B)** cations, bis-(2,4,4-trimethylpentyl)phosphinate **(C)**, and decanoate **(D)** anions, butyric **(E)**, and lactic **(F)** acids. In panel **(B)** *n* = 6, 8, or 10.

Ionic liquids (ILs) used in the experiments are summarized in Table 1. [C_14_C_6_C_6_C_6_P^+^][BTMPP^−^] and [C_14_C_6_C_6_C_6_P^+^][C_9_COO^−^] are commercial products and [C_n_C_n_C_n_C_1_N^+^][BTMPP^−^] was synthesized by metathesis from precursors [C_n_C_n_C_n_C_1_N^+^][Cl^−^] and BTMPP-H (Blahušiak et al., 2013). Before use, all ILs were conditioned by washing with equal volumes of the aqueous solution of 0.5 and eventually 0.15 kmol.m^−3^ NaOH and then typically more than 10 times with deionized water until a constant pH value of the aqueous phase was achieved. Also, the precursors were conditioned. The structural formulas of IL anions and cations are shown in Figure 2.

**Table 1:** Ionic liquids used.

**IL short name**	**IL full name**	**Trade name**	**Producer**	**Purity** **wt. %**
[C_14_C_6_C_6_C_6_P^+^][BTMPP^−^]	tetradecyltrihexylphosphonium bis-(2,4,4-trimethylpentyl) phosphinate	Cyphos IL-104	Cytec (Canada)	95
[C_14_C_6_C_6_C_6_P^+^][C_9_COO^−^]	tetradecyltrihexylphosphonium decanoate	Cyphos IL-103	Cytec (Canada)	95+
[C_n_C_n_C_n_C_1_N^+^][BTMPP^−^]	trialkylmethylammonium bis-(2,4,4-trimethylpentyl) phosphinate, *n* = 6, 8, or 10	–	Synthetized (Blahušiak et al., 2013)	89+

Lactic acid (LA, Figure 2F) extra pure was purchased from Merck (Germany) as a 90 wt. % aqueous solution. Before use it was five times diluted with deionized water and boiled under the total reflux of distillate for >5 h to split the acid dimer. Butyric acid (BA, Figure 2E) with the purity of >99 wt. % was also supplied by Merck. Dodecane with the purity of above 98 wt. % (Fluka, Switzerland) was used as a diluent.

All experiments were done at the temperature of 298.15 K. Liquid–liquid equilibrium experiments were carried out using 4, 10, or 25 cm^3^ vials according to the final volume of the quaternary two-phase system (acid + water + IL + dodecane). The equilibrium was reached after more than 10 h in a rotational shaking water bath (GFL, Germany). The intensity of shaking was optimized to ensure the dispersion of phases and to avoid the formation of stable emulsion.

Analysis of LA and BA in the aqueous phases was done by capillary electrophoresis using an analyzer EA 100 (Villa, Slovakia). In experiments with [C_14_C_6_C_6_C_6_P^+^][C_9_COO^−^], BA was analyzed on an Agilent Technologies 1260 Infinity HPLC system (USA). More detailed descriptions of used chemicals, experimental methods and analyses are provided in previous works (Marták and Schlosser, 2007, 2016).

## Results and Discussion

Differences between the new model of liquid-liquid equilibrium and the previous one used in papers (Marták and Schlosser, 2007, 2008) are explained in papers (Marták and Schlosser, 2016). Briefly, the main differences are that the stability constants of the previous model were the equilibrium constants of (*p*, 1) complex formation from its free components (acid and IL) but in the new model, they express the equilibrium between complexes (*p* – 1, 1) and (*p*, 1). The advantage is that constant *K*_*p*_ now characterizes the stability of only one acid-IL or acid-acid bond (Equations 1 and 2) and it does not include all bonds of the complex. Second difference is that at higher acid loadings of IL, all complexes are considered (Equation 5) and not only those selected by the fitting method as it is in an older model.

**Figure 3:**
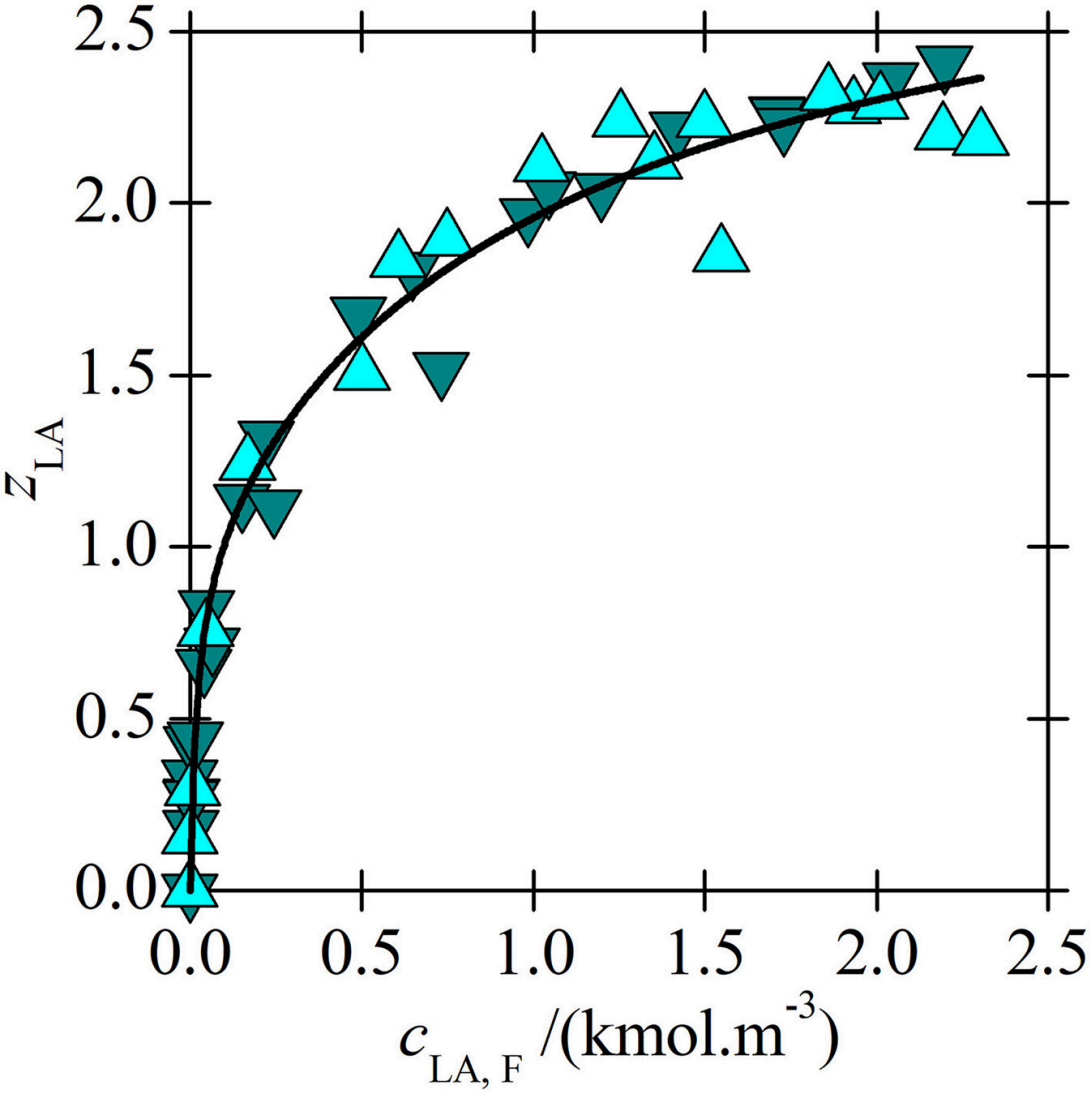
Loading of [C_14_C_6_C_6_C_6_P^+^][BTMPP^−^] by LA as a dependence on the aqueous concentration of undissociated LA in systems (water + 70 wt. % IL in dodecane) (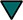) and (water + undiluted IL) (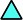). The line was correlated according to the model based on experimental data in paper (Marták and Schlosser, 2007).

**Figure 4:**
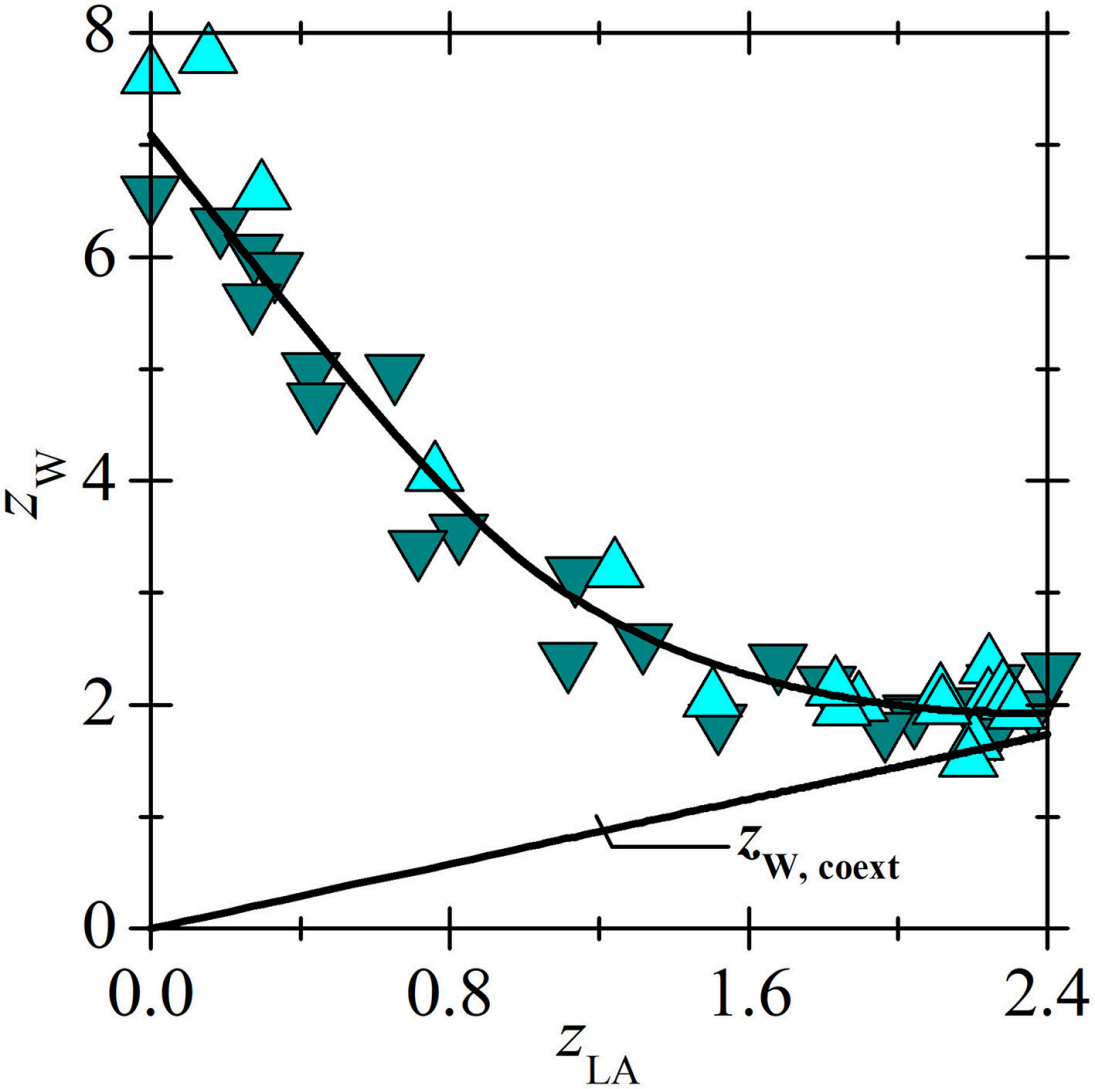
Loading of [C_14_C_6_C_6_C_6_P^+^][BTMPP^−^] by water vs. loading by LA in the same systems as in Figure 3. Lines represent the values calculated according to the model based on experimental data in paper (Marták and Schlosser, 2007). For *z*_W. coext_ see Equation (9).

**Figure 5:**
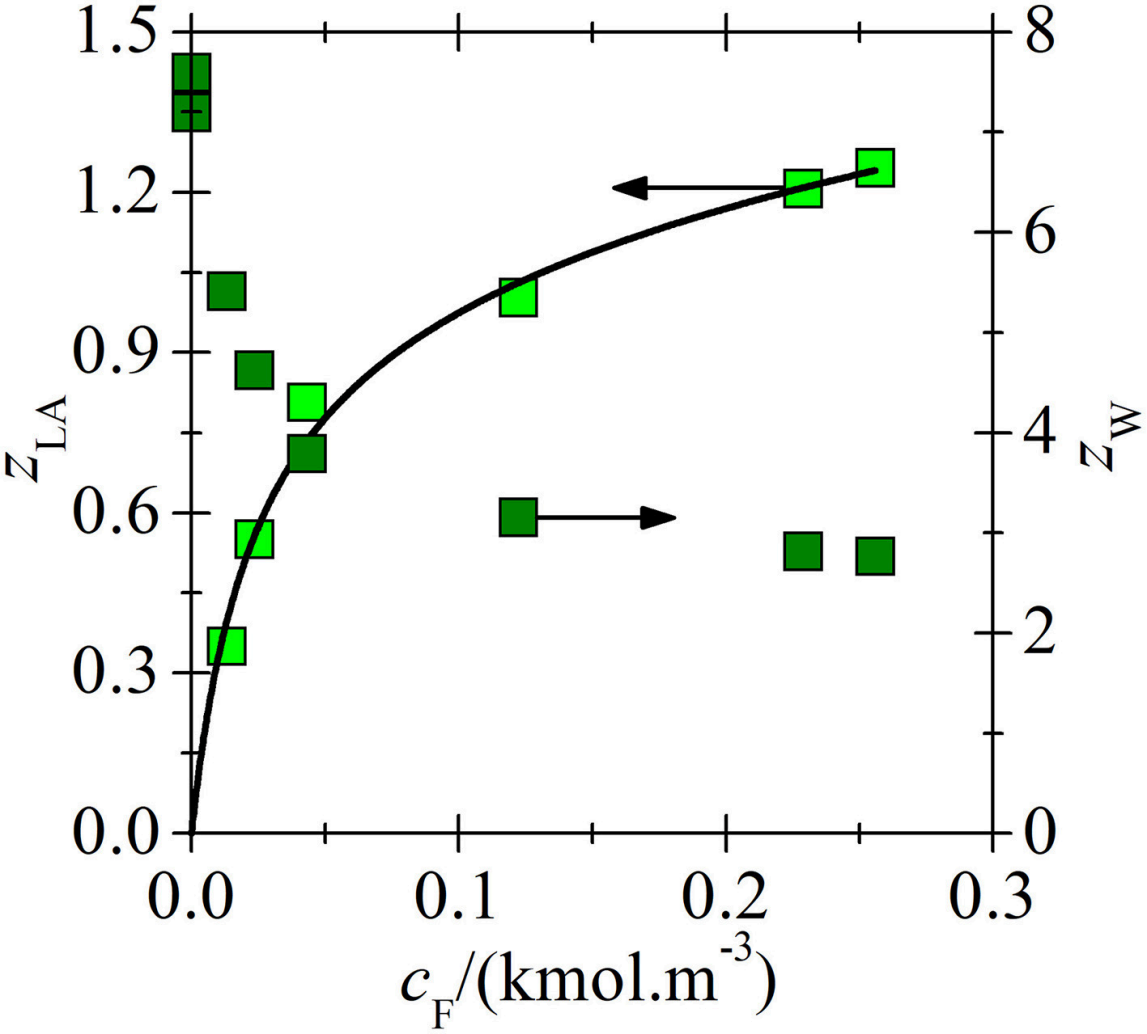
Loading of [C_14_C_6_C_6_C_6_P^+^][C_9_COO^−^] by LA (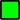) and water (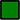) vs. equilibrium LA concentration of undissociated LA in the aqueous phase in system (water + undiluted [C_14_C_6_C_6_C_6_P^+^][C_9_COO^−^]). The line correlates experimental data according to the model. Loading of IL by water was not correlated because of insufficient number of data points.

Application of the new model on experimental data for lactic acid (LA) and [C_14_C_6_C_6_C_6_P^+^][BTMPP^−^] is shown in Figures 3, 4. After consideration, the data for a 30 % [C_14_C_6_C_6_C_6_P^+^][BTMPP^−^] solution previously published in papers (Marták and Schlosser, 2007, 2017) were not used because of the formation of a dodecane-rich third phase as shown in paper (Marták and Schlosser, 2017). New data on LA extraction with [C_14_C_6_C_6_C_6_P^+^][C_9_COO^−^] are presented in Figure 5.

Even though lactic acid is stronger (p*K*_*a*_ = 3.86 at 298 K) than butyric acid (BA, p*K*_*a*_ = 4.82 at 298 K) it has been shown that its affinity to basic amine extractants is much lower compared to that of BA (Procházka et al., 1994; Sabolová et al., 2001). The same applies also for phosphonium ILs as it has been reported in the previous works (Marták and Schlosser, 2007, 2016, 2017) as well as for the new data on LA extraction by [C_14_C_6_C_6_C_6_P^+^][C_9_COO^−^] (Figure 5).

For LA, the values of constant *K*_1_ (Table 2) representing the stability of acid-IL interaction includes mainly the strength of polar interactions between the acid and IL and the contribution of hydrophobic interactions is rather insignificant since LA contains only one methyl group. For BA, constants *K*_1_ are by more than one order of magnitude higher, which indicates rather high contribution of hydrophobic interactions between the acid and IL in complex (1, 1). Therefore, for BA extraction, *K*_1_ should be a lumped constant, which includes both polar interactions represented by the strength of the BA-IL H-bond as well as the hydrophobic interactions. The same follows also for *K*_*p*_ for *p* > 1.

**Table 2:** Values of parameters *K*_*p*_, *A, B, C, k, k*_1_, *k*_2_, *k*_1,Wtot_, *k*_2,Wtot_, and *K*_W,coext_ resulting from the fitting of the data on (Liquid-Liquid) equilibrium in the system (IL + dodecane + acid + water) by the studied model.

**Acid**	**LA**	**LA**	**BA** **(Marták and Schlosser, 2016)**	**BA** **(Marták and Schlosser, unpublished manuscript)**	**BA**
**IL**	**[C_14_C_6_C_6_C_6_P^+^]** **[BTMPP^−^]**	**[C_14_C_6_C_6_C_6_P^+^]** **[C_9_COO^−^]**	**[C_14_C_6_C_6_C_6_P^+^]** **[BTMPP^−^]**	**[C_14_C_6_C_6_C_6_P^+^]** **[C_9_COO^−^]**	**[C_n_C_n_C_n_C_1_N^+^]** **[BTMPP^−^]** ***n* = 6, 8, or 10**
***P***	$\frac{K_{p}}{{\text{m}}^{3}.{\text{kmol}}^{-1}}$	$\frac{K_{p}}{{\text{m}}^{3}.{\text{kmol}}^{-1}}$	$\frac{K_{p}}{{\text{m}}^{3}.{\text{kmol}}^{-1}}$	$\frac{K_{p}}{{\text{m}}^{3}.{\text{kmol}}^{-1}}$	$\frac{K_{p}}{{\text{m}}^{3}.{\text{kmol}}^{-1}}$
1	**51.4**	**47.0**	**1,610**	**524**	**638**
2	**1.89**	**1.77**	**85.4**	**62.2**	**20.6**
3	**0.467**	-	21.1	8.64	21.3
4	-	-	5.03	3.23	5.57
5	-	-	2.36	1.83	2.53
6	-	-	1.58	1.32	1.6
7	-	-	1.28	1.10	1.22
8	-	-	1.15	0.986	1.04
9	-	-	1.08	0.927	0.944
10	-	-	1.05	0.894	0.894
11	-	-	1.03	0.876	0.865
12	-	-	-	0.866	-
13	-	-	-	0.860	-
14	-	-	-	0.857	-
15	-	-	-	0.855	-
16	-	-	-	0.854	-
*A*	-	-	**0.0142**	**−0.160**	**−0.191**
*B*	-	-	**20.7**	**12.2**	**16.0**
*C*	-	-	**−0.639**	**−0.553**	**−0.532**
${\bar{R}}_{BA}^{2}$	0.9740	0.9924	0.9987	0.9978	0.9948
*K*	**7.09**	-	**7.83**	**7.39**	**11.6**
*k*_1_	**2.13**	-	**3.20**	**2.47**	**5.48**
*k*_2_	**0**	-	**0**	**0.880**	**0**
*k*_1_, W_tot_	2.85	-	3.72	3.07	6.22
*k*_2_, W_tot_	1.44	-	1.05 **0.523**^**c**^	2.07	1.48
*k*_*W*_, coext	**0.722**^**b**^	-	**0.410**^**d**^	**0.596**^**e**^	**0.738**^**f**^
${\bar{R}}_{W}^{2}$	0.9419	-	0.9945	0.9726	0.9173

*The fitted parameters are shown in bold. Other parameters were calculated from fitted ones using Equations (5, 11, 12). Temperature: *T* = *298.15*^a^ K, pressure: *P* = *0.10* MPa^a^.*R* squared related to the data on acid extraction (${\bar{R}}_{\text{BA}}^{2}$) and water extraction (${\bar{R}}_{\text{W}}^{2}$) are also presented*.

a *Standard uncertainties u: u(T) = 0.1 K, u_r_(P) = 0.06*.

b *Common value for systems with 70 wt. % IL in dodecane and undiluted IL. Concentrations c_F_ where the differences between K_W,coext_ at various IL concentrations are apparent (higher z_BA_) were not achieved*.

c *Value for undiluted IL*.

d *Value for 60 wt. % IL diluted in dodecane*.

e *Value for undiluted IL. No experiments were done for diluted IL*.

f *Value for undiluted IL. Experiments at high z_BA_ were not available for diluted IL*.

For LA, the values of *K*_1_ are similar for phosphonium ILs with both phosphinate and decanoate anions (Table 2). The same can be said for *K*_2_, indicating that the affinities of LA to both ILs are almost the same. However, for BA, extraction by these ILs, the differences in constants *K*_1_ as well as *K*_2_ are larger, which can be attributed to differences in hydrophobic interactions between the alkyl chains of ILs and BA. The decanoate anion contains linear carbon chain and phosphinate anion includes two branched chains. The presence of branched alkyl chains usually increases the viscosity as a consequence of more intensive van der Waals interactions which can be reflected in higher values of stability constants for extraction of BA by phosphinate IL. Further investigation is needed to verify this idea.

The values of stability constant *K*_*p*_ are the highest for *K*_1_ which are by one to three orders of magnitude higher compared to *K*_*p*_ for higher *p. K*_*p*_ decrease exponentially with the increasing *p* (Equation 5, Table 2). This indicates that the acid-IL bonds are stronger than acid-acid bonds. The strength of the acid-acid bonds decreases as the acid distance from the polar head of IL increases approaching for *p* > 5 a similar value, around the unity, for all observed ILs in BA extraction. Therefore, the stability of acid-acid bonds more distant from the polar head of IL is independent of the IL structure.

**Figure 6:**
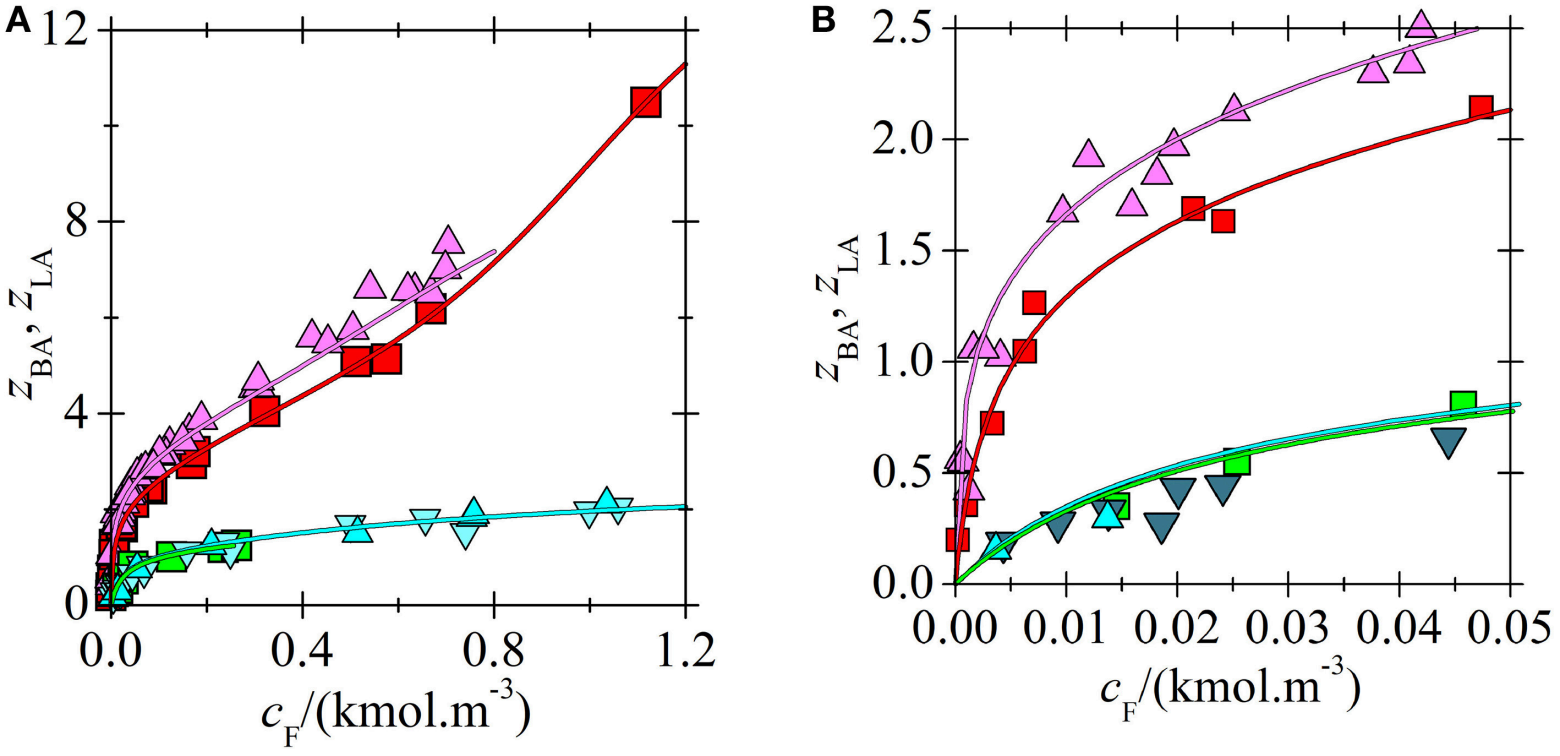
Loading of phosphonium ILs by acids vs. aqueous equilibrium concentration of acid in systems (BA + undiluted [C_14_C_6_C_6_C_6_P^+^][BTMPP^−^]) (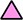) (Marták and Schlosser, 2016), (BA + undiluted [C_14_C_6_C_6_C_6_P^+^][C_9_COO^−^]) (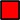) (Marták and Schlosser, unpublished manuscript), (LA + undiluted (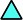) or 70 wt. % (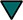) [C_14_C_6_C_6_C_6_P^+^][BTMPP^−^] in dodecane) (Marták and Schlosser, 2007), and (LA + undiluted [C_14_C_6_C_6_C_6_P^+^][C_9_COO^−^]) (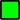). Lines correlate with the experimental data according to the model. **(B)** An extended view of the lower concentration range of **(A)**.

Comparison of acid loadings of phosphonium ILs is shown in Figure 6. Since the overall acid loading of IL for LA exceeds the value of two only moderately even at high LA concentrations (Figure 3), the stability constant of the acid-acid bond in complex (3, 1) formed from complex (2, 1) is very low (Table 2) and hence the bond between the second and the third acid is weak. Due to the high polarity of LA it probably cannot form complexes with more than three molecules of LA.

**Figure 7:**
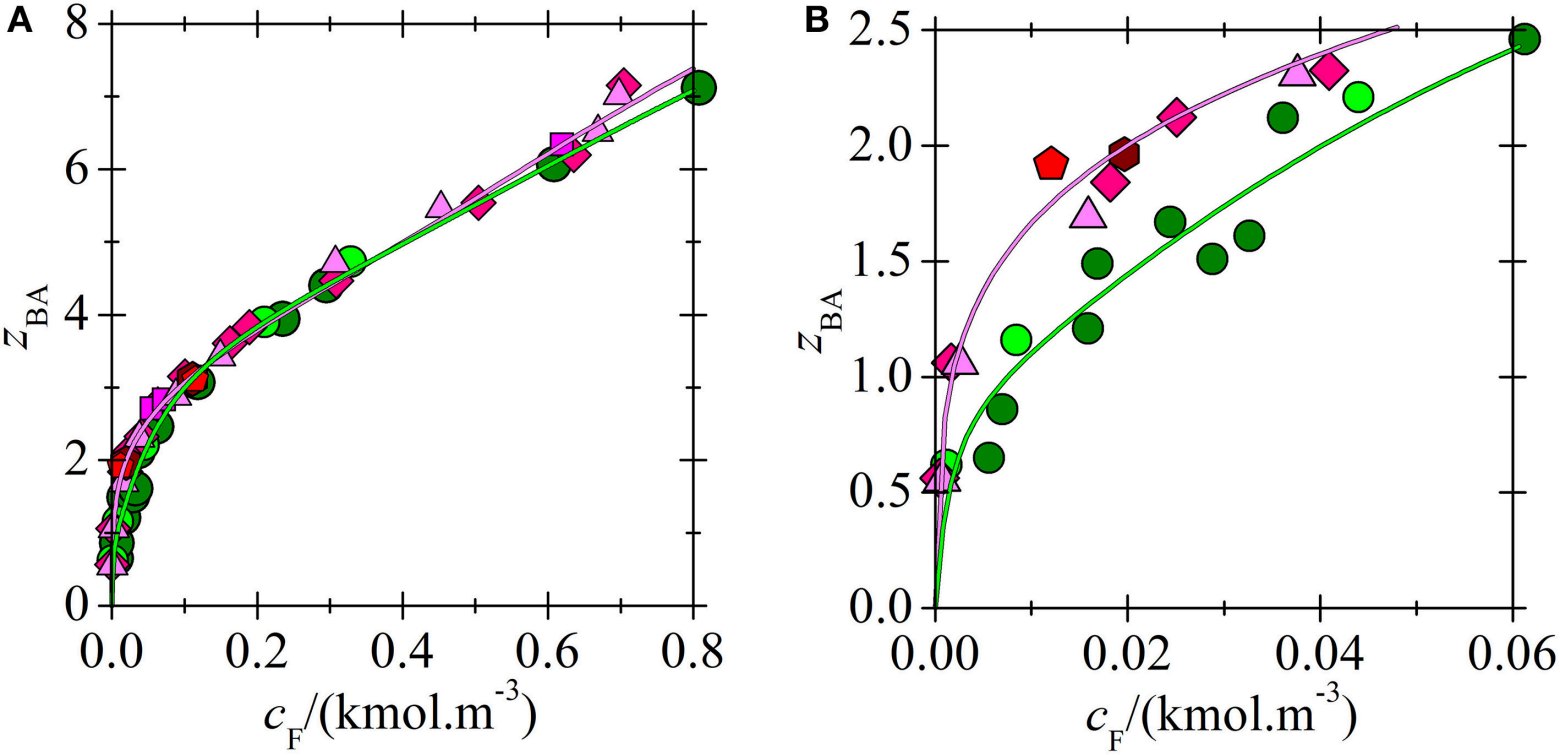
Comparison of BA loading for phosphonium (Marták and Schlosser, 2016) and ammonium (Blahušiak et al., 2013) ILs vs. aqueous equilibrium concentration of acid in two-phase systems (water + IL dissolved in dodecane). Composition of dry solvent phase (wt. % of IL in dodecane): [C_n_C_n_C_n_C_1_N^+^][BTMPP^−^] 70 (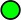), undiluted (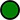), [C_14_C_6_C_6_C_6_P^+^][BTMPP^−^] 40 (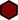), 50 (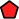), 60 (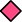), 70 (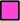), undiluted (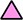). Lines correlate with the experimental data according to the model (green for ammonium and magenta for phosphonium IL). **(B)** An extended view of the lower concentration range of **(A)**.

In contrast to LA, BA with a C_3_ hydrophobic tail can form big complexes with the tested ILs as it follows from the loadings in Figures 6, 7. These complexes are formed by non-competitive mechanism as follows from Equation (2). Even the values of *K*_*p*_ with the highest *p* are about twice higher than *K*_3_ for LA, Table 2. However, due to the high values of constant *K*_1_ (Table 2), i. e. high affinity of ILs to the first BA molecule, the recovery yield of BA from extract with [C_14_C_6_C_6_C_6_P^+^][BTMPP^−^] by distillation in a short-path vacuum evaporator cannot achieve higher value than about 90% at 160°C (Blahušiak et al., 2011, 2012). It requires higher distillation temperature, which can be a problem when considering limit in temperature stability of IL. Much lower values of *K*_1_ for [C_14_C_6_C_6_C_6_P^+^][C_9_COO^−^] with a decanoate anion or for ammonium IL with a phosphinate anion (Table 2) can be advantageous as it is suggested by the preliminary data for [C_14_C_6_C_6_C_6_P^+^][C_9_COO^−^]. In the short path vacuum evaporator with wiped film operating continuously in the same way as in paper (Blahušiak et al., 2011), binary solutions of phosphonium ILs were distilled with 30 wt. % BA at 130°C and pressure of 2 kPa. The recovery of BA from phosphonium ILs with phosphinate and decanoate anions was about 88 and 94%, respectively, proving the advantage of ILs with a decanoate anion in regeneration.

**Figure 8:**
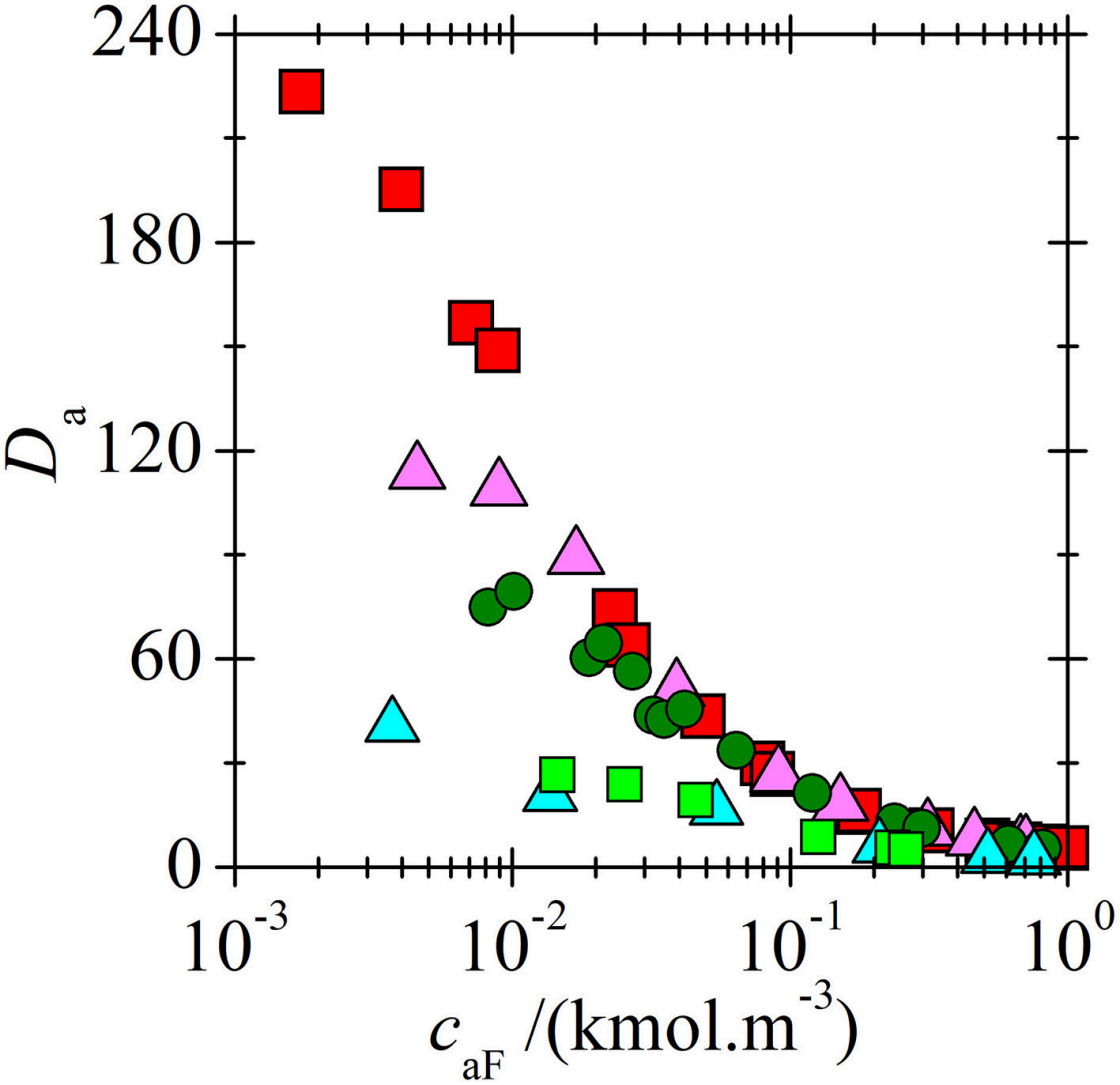
Concentration dependences of acids distribution coefficients in two-phase systems (water + IL) for BA extracted by [C_14_C_6_C_6_C_6_P^+^][C_9_COO^−^] (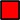) (Marták and Schlosser, unpublished manuscript), [C_14_C_6_C_6_C_6_P^+^][BTMPP^−^]) (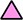) (Marták and Schlosser, 2016), [C_14_C_6_C_6_C_6_P^+^][BTMPP^−^] (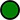) (Blahušiak et al., 2013) and LA extracted by [C_14_C_6_C_6_C_6_P^+^][C_9_COO^−^] (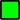) and [C_14_C_6_C_6_C_6_P^+^][BTMPP^−^] (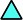) (Marták and Schlosser, 2007).

The correlation between *K*_*p*_ values and extraction capability of ILs is not direct. For example, in extraction of BA *K*_1_ decreases in order of ILs [C_14_C_6_C_6_C_6_P^+^][BTMPP^−^] > [C_n_C_n_C_n_C_1_N^+^][BTMPP^−^] > [C_14_C_6_C_6_C_6_P^+^][C_9_COO^−^] (Table 2). However, at low BA concentrations where the complex (1, 1) is mostly formed, the distribution coefficients indicating the extraction capability decreases in the order [C_14_C_6_C_6_C_6_P^+^][C_9_COO^−^] >[C_14_C_6_C_6_C_6_P^+^][BTMPP^−^] > [C_n_C_n_C_n_C_1_N^+^][BTMPP^−^] as shown in Figure 8 so that IL providing lowest *K*_1_ has the highest extraction capability. Such paradox can be explained as follows: for example, the densities and water mass fractions in water saturated [C_n_C_n_C_n_C_1_N^+^][BTMPP^−^] and [C_14_C_6_C_6_C_6_P^+^][C_9_COO^−^] are very similar (about 0.9 g.cm^−3^ and 16%). However, the molecular weight of [C_14_C_6_C_6_C_6_P^+^][C_9_COO^−^] (655.1 g.mol^−1^) is lower compared to [C_14_C_6_C_6_C_6_P^+^][BTMPP^−^] (773.3 g.mol^−1^) so that it has higher molarity. Due to higher molarity of [C_14_C_6_C_6_C_6_P^+^][C_9_COO^−^] the distribution coefficient can be higher although *K*_1_ is lower. Comparing Table 2 and Figure 8 the same can be said also for extraction of LA. In the future research the structure of IL providing low *K*_1_, high extraction capability and low viscosity should be found. However, this is not an easy task because ILs composed from small ions providing higher molarity as well as low-viscosity ILs are usually more polar, and hence more soluble in water.

**Figure 9:**
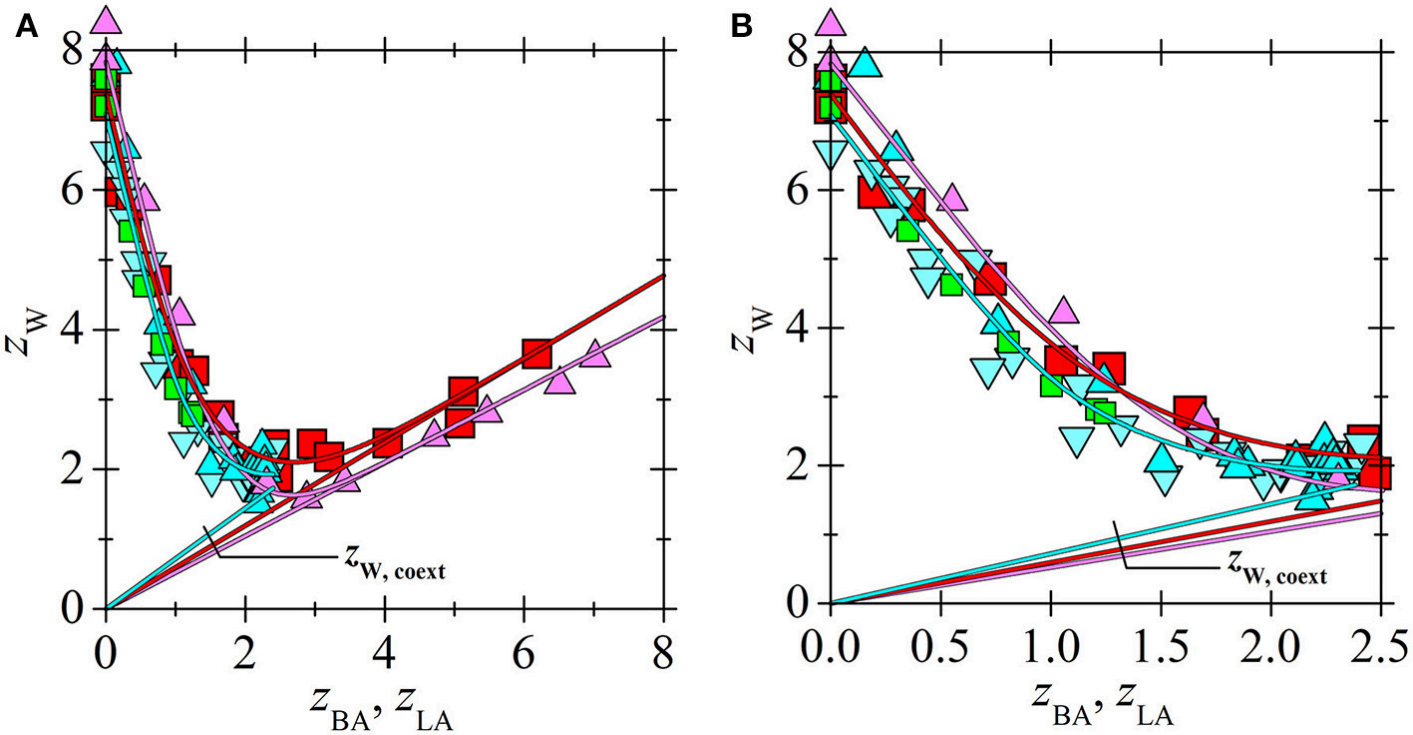
Loading of phosphonium ILs by water vs. loading of IL by acid for the same systems as in Figure 6. (BA + undiluted [C_14_C_6_C_6_C_6_P^+^][BTMPP^−^]) (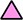) (Marták and Schlosser, 2016), (BA + undiluted [C_14_C_6_C_6_C_6_P^+^][C_9_COO^−^]) (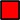) (Marták and Schlosser), (LA + undiluted (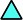) or 70 wt. % (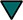) [C_14_C_6_C_6_C_6_P^+^][BTMPP^−^] in dodecane) (Marták and Schlosser, 2007) and (LA + undiluted [C_14_C_6_C_6_C_6_P^+^][C_9_COO^−^]) (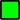). Diluent was dodecane. For *z*_W. coext_ see Equation (9). Lines correlate with the experimental data according to the model. **(B)** The extended view of the lower acid loading range of **(A)**.

**Figure 10:**
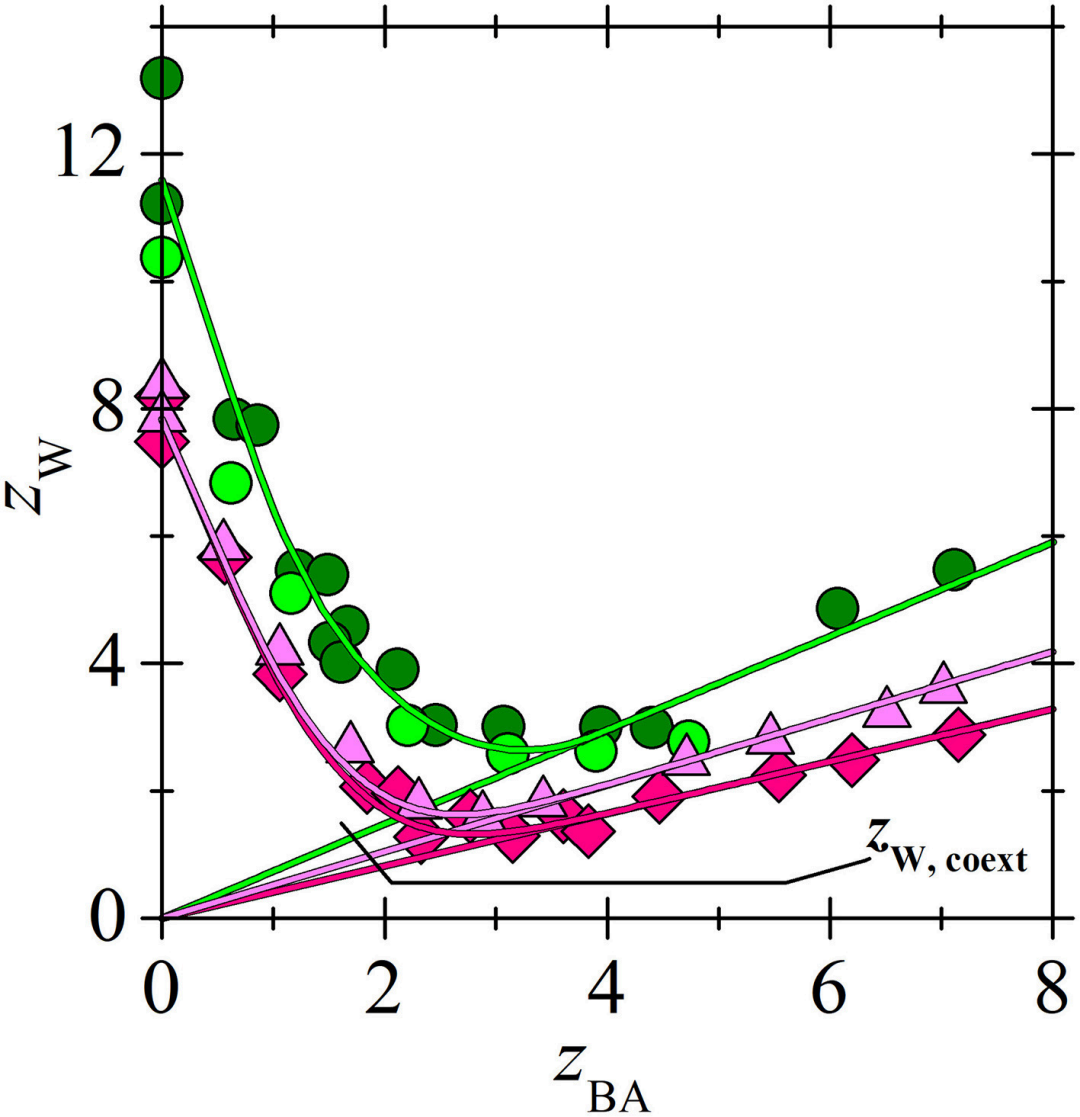
Comparison of water loading of IL for phosphonium (Marták and Schlosser, 2016) and ammonium (Blahušiak et al., 2013) ILs vs. BA loading of IL for the selected systems in Figure 7. Composition of dry solvent phase (wt. % of IL in dodecane): [C_n_C_n_C_n_C_1_N^+^][BTMPP^−^] 70 (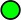), undiluted (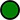), [C_14_C_6_C_6_C_6_P^+^][BTMPP^−^] 60 (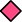), undiluted (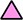). For *z*_W. coext_ see Equation (9). Lines correlate with the experimental data according to the model.

All studied ILs are hydrophobic but they can dissolve large amount of water (Figures 9, 10) (Marták and Schlosser, 2007, 2016; Blahušiak et al., 2013). The differences in water solubility in [C_14_C_6_C_6_C_6_P^+^][BTMPP^−^] for experiments with LA and BA, as follows from Figure 9B and values of constant *k* in Table 2, are probably due to different IL batches from the supplier. The dependence of water loading on the acid loading of ILs is similar for all tested ILs. In Figure 10 it is shown that water content in ammonium IL is higher in the whole range of acid loadings of the IL compared to that in phosphonium IL, probably due to the higher polarity of the ammonium cation caused by higher charge density on nitrogen compared to phosphorus.

At lower acid concentrations when the acid-IL complexes are formed mainly by competitive mechanism (Equation 1) acid competes with water for H-bond sites situated on carboxylate or phosphinate oxygens of IL anions. The formation of H-bonds between water and oxygen located on IL anion has been already suggested and modeled by atomistic simulation, e.g., for tetraalkylphosphonium bis(oxalato)borate ionic liquid (Wang et al., 2015). With the increasing acid content, water is substituted with acid (Figures 9, 10). Typically, about half of the water amount is released from water saturated IL when complex (1, 1) is formed as it follows from the comparison of *k* and *k*_1,Wtot_ in Table 2 (see also Equation 11). In BA extraction, after reaching the minimum of IL loading by water, (*p*, 1) complexes are formed by non-competitive mechanism (Equation 2) and Figure 9 documents that water content in the solvent phase increases linearly with the increasing acid loading of the IL. This increase indicates that such water is not associated directly with the IL (both H-bonding sites on IL anion are occupied by acid) but with the acid (also by H-bond), and the water to acid molar ratio is given by constant *K*_W,coext_ as it follows from Equation (9) (Marták and Schlosser, 2016). As it can be seen in Figure 9, the dependence of water loading on IL loading cannot reach the region of increasing water loading in LA extraction due to low acid loading of the IL. Anyway, it is assumed that similarly to BA, also extracted LA is hydrated and constant *K*_W,coext_ was calculated as shown in Table 2. A comparison of the results with LA and BA extracted by [C_14_C_6_C_6_C_6_P^+^][BTMPP^−^] shows that the value of *K*_W,coext_ is higher for LA than for BA, which can be attributed to the higher polarity of LA.

The differences between coefficients *k*_*p*_ and *k*_*p*__, Wtot_ enable us to consider the distribution of water in individual complexes (Equations 11 and 12). As follows from Equation (11) and Table 2, in the complex of [C_14_C_6_C_6_C_6_P^+^][BTMPP^−^] with LA (1, 1) there are typically two molecules of water bound to the IL and one to LA. For BA extraction, complex (1, 1) with [C_14_C_6_C_6_C_6_P^+^][BTMPP^−^] contains about three molecules of water bound to the IL and only half water molecule was left to bond with the acid. This can be explained by the association of complexes via a water bridge as suggested in previous work (Marták and Schlosser, 2016). In BA extraction with [C_14_C_6_C_6_C_6_P^+^][C_9_COO^−^], a comparison of the values of *k*_1_, *k*_1,Wtot_, and *K*_W,coext_ showed that complex (1, 1) contains probably two or three water molecules associated with the IL and one water molecule can form a bridge between the complexes. In BA extraction with [C_n_C_n_C_n_C_1_N^+^][BTMPP^−^], complex (1, 1) contains about five or six water molecules associated with the IL and one with the acid.

A comparison of *k*_*p*_ and *k*_*p*__, Wtot_ also provides that complex (2, 1) contains only water associated with acids in all tested systems except for BA with [C_14_C_6_C_6_C_6_P^+^][C_9_COO^−^], where constant *k*_2_ (Equation 12, Table 2) has a non-zero value. The value of *k*_2_ close to unity suggests one water molecule associated with IL. Hypothetical structure of the (BA + [C_14_C_6_C_6_C_6_P^+^][C_9_COO^−^]) complex (2, 1) is shown in Figure 2 (Marták and Schlosser, unpublished manuscript).

## Conclusions

New mechanism and a model of reactive extraction of carboxylic acids by hydrophobic ionic liquids (ILs) published in Marták and Schlosser (2016) was tested on a wider set of experimental data published earlier (Marták and Schlosser, 2007; Blahušiak et al., 2013) and new equilibrium data on liquid-liquid extraction of butyric and lactic acids (BA and LA) from aqueous solutions by two phosphonium and one ammonium ionic liquids (ILs). The model describes experimental data for five systems with a good fit which indicates its more general validity. It allows deeper understanding of regularities in carboxylic acid extraction by ILs.

The values of stability constants *K*_1_ of acid-IL bond are one to three orders of magnitude higher compared to *K*_*p*_ with higher *p. K*_*p*_ decreases exponentially with the increasing *p*. Therefore, acid-IL bonds are stronger than acid-acid bonds. Differences in the stability of acid-IL bonds for BA and LA can be attributed to hydrophobic interactions which almost do not occur in LA extraction. Stability of acid-acid bonds with *p* > 5 are not sensitive to the IL structure.

The values of stability constants *K*_*p*_ of acid-IL bonds are more than one order of magnitude lower for LA compared to BA, despite LA being a stronger acid than BA. In addition to H-bonding, hydrophobic interactions of BA with IL contribute to the overall value of *K*_*p*_ which is lumped constant.

The loading of IL by LA exceeds the value of two only moderately so that the stability constant of acid-acid bonds between acids in complex (3, 1) is very low. Due to the high polarity of LA it likely does not form complexes with *p* > 3. BA is more hydrophobic than LA so that it forms complexes with *p* > 3.

Substitution of an anion or cation in the IL influences the values of stability constants *K*_*p*_, especially in BA extraction. The affinity of LA to both phosphonium ILs is almost the same since the values of appropriate stability constants are similar. BA has higher affinity to IL with [BTMPP^−^] which could be attributed to the more intensive van der Waals forces with the anion containing branched alkyls.

The correlation between the value of *K*_1_ characterizing the affinity of acid to IL and extraction capability of IL is not proportional but more complex. For example, the value of *K*_1_ for [C_14_C_6_C_6_C_6_P^+^][BTMPP^−^] is higher than for [C_14_C_6_C_6_C_6_P^+^][C_9_COO^−^] but the extraction capability characterized by distribution coefficient is for [C_14_C_6_C_6_C_6_P^+^][C_9_COO^−^] higher due to its higher molarity.

The recovery yield of BA in short-path vacuum distillation from an extract with [C_14_C_6_C_6_C_6_P^+^][C_9_COO^−^] or [C_n_C_n_C_n_C_1_N^+^][BTMPP^−^] can be higher compared to that with [C_14_C_6_C_6_C_6_P^+^][BTMPP^−^] since the values of stability constant *K*_1_ in BA extraction by these ILs are much lower. In this regeneration process free acid is recovered instead of acid salts in classical processes what is a great advantage.

## Data Availability

The datasets generated for this study are available on request to the corresponding author.

## Author Contributions

JM and ŠS: model development and testing, manuscript writing; JM: measurement of equilibrium data.

### Conflict of Interest Statement

The authors declare that the research was conducted in the absence of any commercial or financial relationships that could be construed as a potential conflict of interest.
